# End-to-end approach to ensuring equitable access to multipurpose prevention technologies in low- and middle-income countries

**DOI:** 10.3389/frph.2023.1238813

**Published:** 2023-08-24

**Authors:** Anne-Isabelle Cameron, Cherise Scott, Carmen Pérez-Casas, Taryn Barker

**Affiliations:** ^1^ Department of Strategy, Unitaid, Geneva, Switzerland; ^2^ Department of Sexual and Reproductive Health and Rights, Children's Investment Fund Foundation, London, United Kingdom

**Keywords:** multipurpose prevention technologies (MPTs), equitable access, low- and middle-income countries (LMICs), HIV, contraception, sexually transmitted infections (STIs)

## Introduction

With less than half of the 15-year Sustainable Development Goal (SDG) agenda remaining, progress toward the goal of ensuring healthy lives and promoting wellbeing for all has slowed, stalled, and in some cases reversed. UNAIDS has targets of 95% of people at risk of human immunodeficiency virus (HIV) infection using appropriate, prioritized, person-centered and effective combination prevention options and 95% of women of reproductive age having their HIV and sexual and reproductive health service needs met by 2025 (1). However, in 2021, there were 1.5 million new HIV infections, far from the 2025 target of 370,000 (2); 121 million unintended pregnancies, nearly half of all pregnancies globally (3); and 374 million new infections with one of four curable sexually transmitted infections (STIs) (4), compounding the risk of HIV acquisition. Most of this burden is borne by low- and middle-income countries (L/MICs). The COVID-19 pandemic pushed progress further off-track. For example, an estimated 12 million women were unable to access family planning services due to the pandemic, resulting in 1.4 million unintended pregnancies (5). With additional factors such as climate change driving migration and conflict, and the cost-of-living crisis constraining budgets, there is even greater need for streamlined services and improved, affordable technologies to meet diverse health needs across different contexts (6).

Novel forms of multipurpose prevention technologies (MPTs) are a game-changing innovation for the simultaneous prevention of unintended pregnancy, HIV, and/or STIs. By addressing multiple health needs at once, MPTs have the potential to better meet the needs of the populations they seek to serve and accelerate progress toward global goals. However, technological innovation alone is insufficient to achieve global impact. Addressing timely and equitable access is key for novel technologies to reach populations in need and realize their full potential. There is an opportunity to learn from other technologies that have emerged in recent decades. On average, it has taken eight to ten years for HIV medicines approved by the United States Food and Drug Administration (US FDA) to become accessible in L/MICs (7). With the urgent need to accelerate progress toward SDG3^1^ (8), we cannot afford preventable mortality and morbidity caused by this lag in access.

## E2E approach to access in L/MICs

**Figure 1:**
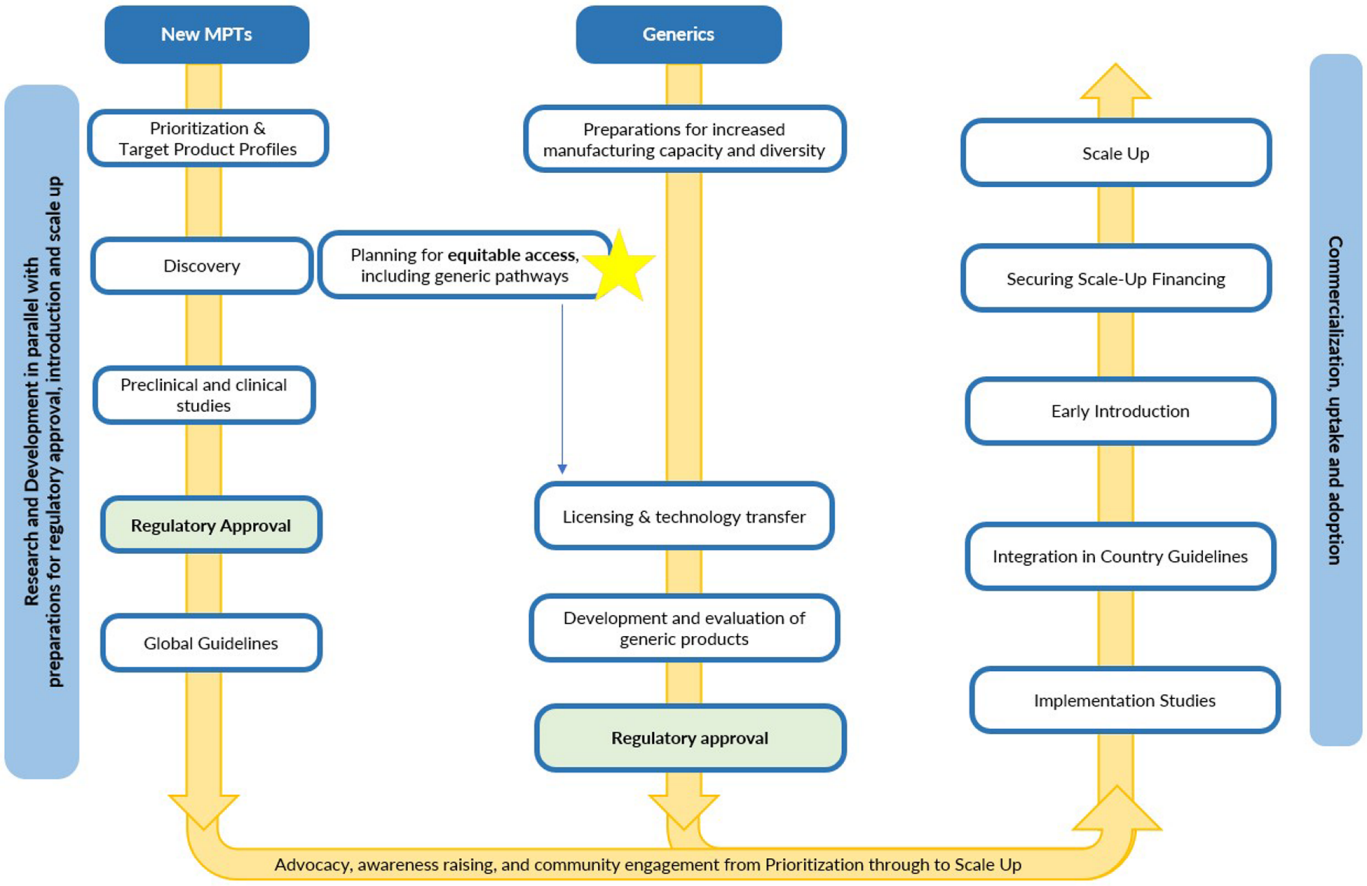
Planning an E2E approach.

An end-to-end (E2E) approach in the context of addressing health innovations aligns relevant stakeholders across sectors, including product developers, generic manufacturers, funders, regulators, health agencies, governments and communities, on a common strategy to bring a product from research and development to uptake and adoption by communities (Figure 1). An E2E approach has a holistic view of the actions and commitments necessary to facilitate equitable access and must be initiated at the earliest stages of product development. Waiting until products reach the market to plan for equitable access often leaves it too late to address the root causes of high prices and slow commercialization in L/MICs (7). The full potential of any given MPT can best be realized if an E2E approach is taken to ensure the conditions to achieve equitable access are established rapidly—including in advance of products being available—and in a sustainable manner.

An E2E approach goes beyond advancing products in pipeline and looks ahead at the roadmap to suitable, affordable and quality supply for accelerated introduction and adoption. This approach prevents and removes potential roadblocks associated with intellectual property, facilitates timely regulatory approvals; anticipates supply pathways; coordinates with country stakeholders for integration into guidelines; and maximizes the engagement of affected communities (6). The following sections outline key considerations for an E2E approach for MPTs, divided into the phases before, during and after regulatory approval.

Figure 1 is a linear depiction of the steps required, however in practice many of these steps overlap to expedite timelines.

### Pre-regulatory approval

The research and development phase for MPTs is a critical time for developers to consider end-user preferences and to chart a product's access pathways to ensure the final product will be both acceptable and accessible to all who could benefit. Investors will similarly need to prioritize which products to support based on end-user preferences and access profiles. User preferences vary widely, however some studies have shown a general preference for discreet and familiar provider-administered long-acting products, such as injectables and implants (9). That said, as one MPT will not meet the needs or preferences of all potential users, this prioritization needs to be balanced with the objective of having a range of MPTs available to enable choice. Condoms are the only MPT currently available, and while they have been and continue to be pivotal in the global agenda to prevent HIV, STI, and unintended pregnancy, they do not meet the preferences of all potential users. This speaks to the need to have a range of different MPTs available, and to establish feedback mechanisms from users to inform the design of future products.

Identifying and mitigating potential barriers to access for end-users, such as affordability and availability, also needs to be initiated by product developers and those financing these innovations in parallel with research and development efforts. The complexity of combining active pharmaceutical ingredients and the technologies involved in MPTs may imply cost differences with the standard-of-care products they aim to replace, especially for formulations with extended release, and may also limit the number and location of manufacturers able to produce them (9). Cost-effectiveness estimates and potential impact need to be considered from early stages to inform which MPTs in the pipeline are prioritized, and developers may need to be incentivized by global health funders to investigate cost-reduction strategies. Global health funders can play an important role by embedding access terms, such as sharing of intellectual property, into funding agreements for research and development or considering market shaping support, such as guaranteeing minimum volumes of procurement in early introduction phases (see Box 1).

Box 1 **Long-acting contraceptives have demonstrated the potential for partnerships to catalyze a healthy market for L/MIC access.**

The Implants Access Program (IAP) was a partnership between industry, procurers, donors, and implementers that began in 2012 with volume guarantees for two contraceptive implants, resulting in a price reduction of approximately 50% (10). This, in combination with efforts to improve supply chain coordination, service delivery, and knowledge and awareness, was key in facilitating rapid scale-up in L/MICs (11). Between 2010 and 2018, there was a tenfold increase in implant procurement in the 69 lowest income countries with 27 million women using implants in L/MIC in 2019 (11, 12, 13). Effective collaboration at global and country levels contributed to institutionalizing mechanisms to maintain lower prices for long term sustainability (11).

Box 2 **Lessons learned from delayed access to HIV treatment and prevention.**

There are many lessons that can be learned from antiretroviral-based therapies and preventatives for HIV. When highly-active antiretroviral therapy first emerged in 1996, prohibitively expensive prices meant that AIDS-related mortality declined by 75% in three years in the United States but continued to rise in L/MICs. It wasn't until 2001, when lower-cost generic versions became available, that mortality started to decline in L/MICs (19). Similar patterns were seen with the emergence of fixed-dose combination therapies, which took nine years following approval by the US FDA to have generic versions available in L/MICs (19).

Daily oral HIV pre-exposure prophylaxis (PrEP) also faced delays reaching L/MIC populations after initial regulatory approval by the US FDA in 2012. By 2016, the US had nearly 100,000 PrEP initiations (virtually all of the 102,446 initiations worldwide), whereas South Africa had 380 (20). It wasn't until 2021 that South Africa and the US had reached roughly the same number of initiations (approximately 200,000) (20). This was largely due to challenges related to acceptability of the product among potential users and providers, low community awareness and motivation to seek preventative services, and health system bottlenecks (21). The experience of oral PrEP highlights the importance of early consideration of user preferences, advanced demand generation, and planning for post-approval activities that are designed and coordinated to inform decision-making at the national and global levels (21).

Intellectual property may also limit who can manufacture MPTs, especially considering the additional intellectual property involved compared to single-indication products. Early planning by developers to establish licensing mechanisms that enable a generic manufacturing base will not only help overcome intellectual property barriers and improve supply security; it will also enable greater affordability. For example, the Medicines Patent Pool (MPP), an organization founded by Unitaid, employs a model that facilitates non-exclusive, voluntary licensing of life-saving medicines for L/MICs (14). While MPTs would require special considerations for voluntary licensing, this model could help address such access barriers. Engagement between industry partners and MPP prior to regulatory approval accelerates access once approval is obtained. Voluntary licenses may not enable access for all L/MICs, however, and other mechanisms may need to be explored to overcome intellectual property barriers (15)^2^.

### Planning for regulatory approval

Early planning is necessary to ensure regulatory pathways do not cause delays in access to MPTs. Regulatory requirements for MPTs are yet to be clearly defined, which may cause challenges for MPT developers as they are planning what data to generate for approvals. There have been reviews of key regulatory guidance documents and their applicability to MPTs, however there will not be one single regulatory pathway for MPTs (9). Regulatory approval requirements often differ across Stringent Regulatory Authorities and national regulators in L/MICs, and requirements will also vary across MPTs depending on the components. For example, the Dual Prevention Pill under development only requires a bioequivalence study, rather than Phase 3 safety and efficacy trials because both oral dose products are separately approved and their drug-drug interactions well-studied (16).

Developers and regulators are therefore encouraged to engage early on in these discussions, so that approval processes can be executed without delay once products in the pipeline reach this stage. Developers are also encouraged to engage with the World Health Organization (WHO) technical areas regarding guidance recommendations; and the WHO Prequalification program (17) including the WHO Collaborative Procedure for Accelerated Registration (18), to accelerate access to and registration of critical quality-assured products in L/MICs. It is generally expected that developers widely register and supply quality-assured products soon after a recommendation or approval is obtained by a normative body and/or regulatory entity.

### Planning for post-regulatory introduction and scale up

In order to have rapid introduction and scale up once approved, both supply and demand side strategies need to be considered including policy and implementation guidance (see Box 2). MPTs will break the mold of the silos that healthcare systems typically operate in. Supply chains for family planning and HIV commodities are currently disconnected, and financing and procurement streams are typically separated by governments and donors as well. Establishing the budget, procurement mechanisms, and supply and delivery pathways for MPTs will require collaboration among diverse stakeholders, including industry, governments, global health funders, and procurement agencies. This will have major implications for the scope of responsibilities of health departments as MPTs become available, ranging from monitoring and evaluation to health provider training. In addition to the adaptations country health systems will need to make to integrate MPTs, country-specific efforts will also be required to obtain national market authorization and guidelines for sustainable implementation in national programs. Implementation research in early adopter countries will play a critical role in generating real-world evidence to inform recommendations at the national and international level and enable broader scale up. In parallel with research, MPT awareness-raising by and with policymakers, medical associations and advocates through convenings, publications and interest group formations can accelerate uptake once products achieve government commitment to scale (9).

## Discussion

Meaningful innovation requires more than new and improved products; it also necessitates finding novel strategies to collaborate and overcome barriers to equitable access for products to have global impact (6). This article highlights the need for a range of stakeholders to collaboratively address product and market characteristics, such as affordability, supply capacity, intellectual property, regulatory pathways, and ease of use, to enable prompt community adoption. While lessons can be learned from existing products, some considerations will be unique to MPTs and it is imperative that the global community align on how to address these and plan far in advance so that MPTs can reach L/MICs without delay once available. Platforms that bring together donors, agencies, governments, affected communities and advocates to ensure an accelerated, sustainable, and collaborative approach is taken to making MPTs equitably accessible will be essential. If planning to remove access barriers does not start early on, there will be a myriad of challenges impeding timely uptake and scale in L/MICs. As the pipeline of MPTs continues to grow and advance, now is the time to plan for an E2E approach to equitable access.

## Footnotes

^1^Sustainable Development Goal 3 is to ensure health lives and promote well-being for all at all ages. This includes targets relevant to MPTs such as target 3.3 (fight communicable diseases) and 3.7 (universal access to sexual and reproductive care, family planning and education).

^2^Note that while this resource refers to COVID-19 therapeutics, the mechanisms outlined to overcome intellectual property barriers can be applied to other products.
